# Optimizing ciltacabtagene autoleucel outcomes: real-world experience and best practices from a single academic center

**DOI:** 10.3389/fonc.2026.1841031

**Published:** 2026-07-06

**Authors:** Leyla O. Shune, Zahra Mahmoudjafari, Joseph P. McGuirk

**Affiliations:** Division of Hematologic Malignancies and Cellular Therapeutics, The University of Kansas Medical Center, Kansas, KS, United States

**Keywords:** BCMA-directed CAR-T, ciltacabtagene autoleucel (cilta-cel), institutional experience, relapsed/refractory multiple myeloma, toxicity management

## Abstract

Ciltacabtagene autoleucel (cilta-cel), a B-cell maturation antigen (BCMA)–directed chimeric antigen receptor T-cell (CAR-T) therapy, has transformed the management of relapsed or refractory multiple myeloma and demonstrated long-term, treatment-free remissions in patients (CARTITUDE-1: median overall survival, 60.7 months and 33% of patients with progression-free survival ≥5 years). While pivotal clinical trials have demonstrated unprecedented depth and durability of response, the real-world populations can sometimes diverge from trial-eligible cohorts, with greater age variability, comorbidity burden, functional limitations, and logistic challenges that may impact outcomes. As cilta-cel adoption expands across treatment centers into the community and outpatient settings, generating real-world evidence and best practices is critical to ensuring that the transformative potential of this therapy is realized safely and effectively. This is a single-center report on our experience implementing cilta-cel. We share practical strategies that have supported favorable safety and clinical outcomes across the treatment continuum—beginning with patient selection, baseline risk assessment and pre-infusion optimization, through acute monitoring and management of adverse events, to longer-term follow-up. We highlight the importance of structured workflows, multidisciplinary coordination, early referral pathways, anticipatory guidance, and collaborative care models in achieving safe and effective outcomes. As centers continue to refine cilta-cel implementation, these institutional insights will play a key role in shaping best practices for patients receiving CAR-T therapy.

## Introduction

Patients with triple-class exposed (immunomodulatory agents, proteasome inhibitors, and monoclonal antibodies) relapsed/refractory multiple myeloma (RRMM) experience poor survival outcomes with a median overall survival (mOS) of 13.8 months and median progression-free survival (mPFS) of 4.6 months (1–3). The introduction of B-cell maturation antigen (BCMA)-directed chimeric antigen receptor T-cell (CAR-T) therapy, such as ciltacabtagene autoleucel (cilta-cel), delivers deep and durable remissions for patients with RRMM (4, 5).

In the pivotal phase 1b/2 CARTITUDE-1 trial, cilta-cel demonstrated an overall response rate of 97.9% and a stringent complete response of 82.5% at a median follow-up of 27.7 months (6). These data supported the Food and Drug Administration (FDA) approval of cilta-cel in 2022 for patients with RRMM after ≥4 prior lines of therapy (LOT) (6). CARTITUDE-1 demonstrated an mOS of 60.7 months (95% confidence interval [CI], 41.9 months to not estimable; median follow-up, 61.3 months). Notably, 33% of patients remained in remission for ≥5 years after a single infusion of cilta-cel without maintenance therapy, underscoring its curative potential (7).

**Figure 1:**
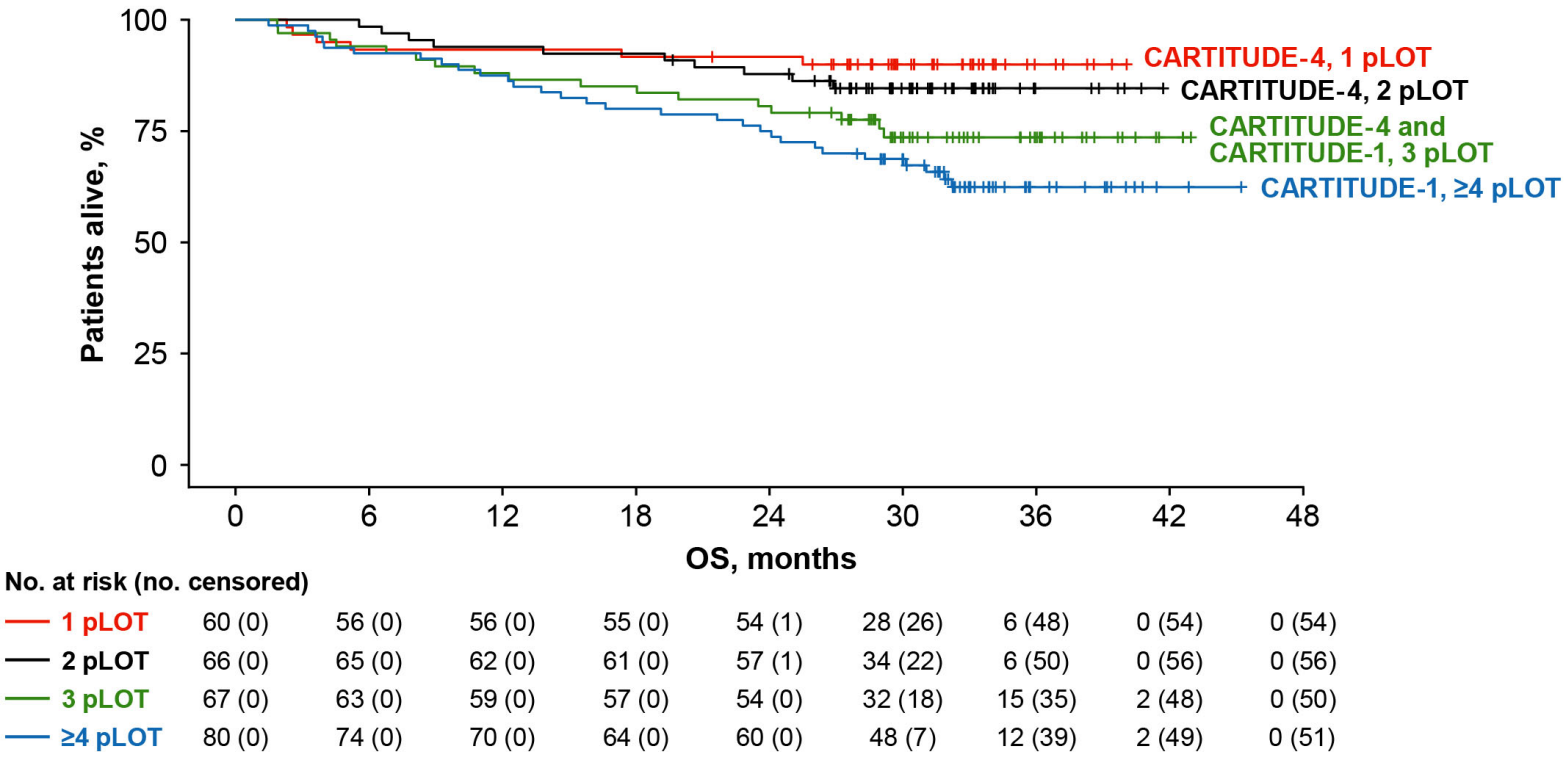
OS by pLOT in CARTITUDE-1 and CARTITUDE-4 studies. Reproduced from: Parekh S, Li K, van de Donk NWCJ, et al. Earlier use of ciltacabtagene autoleucel (cilta-cel) is associated with better immune fitness and stronger immune effects as shown by correlative analysis of peripheral blood and the bone marrow tumor microenvironment (TME) from the CARTITUDE-4 study. Presented at: The American Society of Hematology 2025 Hybrid Congress; December 6-9, 2025; Orlando, FL, USA. Reproduced with permission from the American Society of Hematology (ASH). Median follow-up for CARTITUDE-1 and CARTITUDE-4 studies were 33.4 months and 30.5 months, respectively. OS, overall survival; pLOT, prior line of therapy.

The durable benefit demonstrated with cilta-cel in heavily pretreated patients prompted investigation in earlier LOT (8). Results from the phase 3 CARTITUDE-4 study demonstrated that cilta-cel significantly reduced the risk of disease progression or death compared with standard-of-care (SOC; hazard ratio [HR], 0.26; 95% CI, 0.18-0.38; *P* < 0.001) in patients who received 1–3 prior LOTs, supporting its expanded indication in 2024 for lenalidomide-refractory MM patients after ≥1 prior LOT (9, 10). Longer-term follow-up showed meaningful clinical benefits and improvements in health-related quality of life (QoL) in this population (11). These data, alongside results from the later-line CARTITUDE-1 study, suggest higher survival rates with earlier use of cilta-cel (**Figure 1**) (12). Cilta-cel is currently the only CAR-T therapy to demonstrate a statistically significant OS benefit in patients with RRMM in a randomized phase 3 trial (13). Additionally, in CARTITUDE-4, cilta-cel demonstrated greater cost-effectiveness versus SOC, consistent with real-world cost-per-responder analysis (14, 15). To date, over 10,000 patients globally have received cilta-cel, underscoring the growing experience with this therapy (16).

While pivotal CARTITUDE trials established efficacy and safety, real-world patients often differ from trial populations—typically older with more comorbidities and logistic barriers—with 54% not meeting trial eligibility, yet experiencing favorable efficacy outcomes (17, 18). These differences highlight the importance of real-world evidence in understanding treatment effectiveness. Additionally, institutional learning through which centers refine multidisciplinary workflows, patient selection, and adverse event (AE) management is equally essential for translating clinical trial success into real-world benefit.

This article provides real-world evidence on the use of cilta-cel based on the experience gathered from the treatment of 180 patients with RRMM at the University of Kansas Cancer Center (KUCC) from 2021–2025. It highlights an appropriate framework to successfully achieve optimal outcomes with cilta-cel, describes strategic sequencing, and reinforces early referral as a determinant of treatment eligibility, safety, and long-term benefit.

## Institutional framework and implementation

Successful implementation of cilta-cel requires a robust, multidisciplinary infrastructure designed to manage the logistic, clinical, and operational demands of CAR-T therapy (5, 19). KUCC is the only National Cancer Institute–designated comprehensive cancer center in Kansas serving a large catchment area in the Midwest and is one of the select centers to offer all FDA-approved CAR-T treatments. The CAR-T program is anchored by the Division of Hematologic Malignancies and Cellular Therapeutics, with integration across inpatient and outpatient services. Key partners include the primary clinical team (physicians, advanced practitioners, nurses), apheresis team, cell-processing team, intensive care team, consultative services (neurology and infectious disease), pharmacy, social work (nutritional and counselling teams), and palliative care. A central element is the CAR-T coordination team including dedicated nurse coordinators, advanced practice providers, administrative specialists, social workers, and financial coordinators—who oversee referral intake, travel and lodging arrangements, triage and coordination, financial and insurance authorization, manufacturing timelines, and industry communication.

Early referral from community partners remains one of the significant barriers to CAR-T therapy (20). When referred promptly, ideally at first relapse, patients have the highest likelihood of completing apheresis, avoiding disease-related functional decline, and achieving the performance status needed to be eligible for cilta-cel therapy. Initiating these discussions early, particularly given that cilta-cel is now approved for use as early as first relapse (10), allows sufficient time for patient education and informed decision-making when effective bridging therapy (BT) becomes necessary.

Our workflow follows a defined sequence from referral to infusion: 1. pre-apheresis evaluation; 2. leukapheresis; 3. BT; 4. lymphodepletion chemotherapy; 5. CAR-T infusion; 6. acute phase monitoring (timely transition of patients back to their referring physician); 7. high-fidelity communication and management guidelines for referring providers; 8. immediate-access channels to CAR-T physicians for referring providers. Biweekly interdisciplinary meetings (monitoring frequency, outpatient transition, operationalizing the risk evaluation and mitigation strategy removal) and weekly patient review meetings ensure timely clinical decision-making. Operationally, minimizing vein-to-vein time is a priority (21). Close communication with the specialized manufacturing center and proactive management of potential AEs, including cytopenias, infections, or organ dysfunction, reduce delays. Workflow standardization has enabled a predictable timeline, staff familiarity, and interdepartmental coordination. These logistic structures have contributed to high treatment adherence, reduced unplanned interruptions, and improved the overall experience for patients and caregivers.

## Patient selection and pre-infusion optimization

While pivotal CARTITUDE trials enrolled patients who met strict eligibility criteria related to organ function, treatment history, and comorbidity burden, real-world populations often diverge from those parameters (18). This underscores the need to adapt selection frameworks for real-world settings.

**Figure 2:**
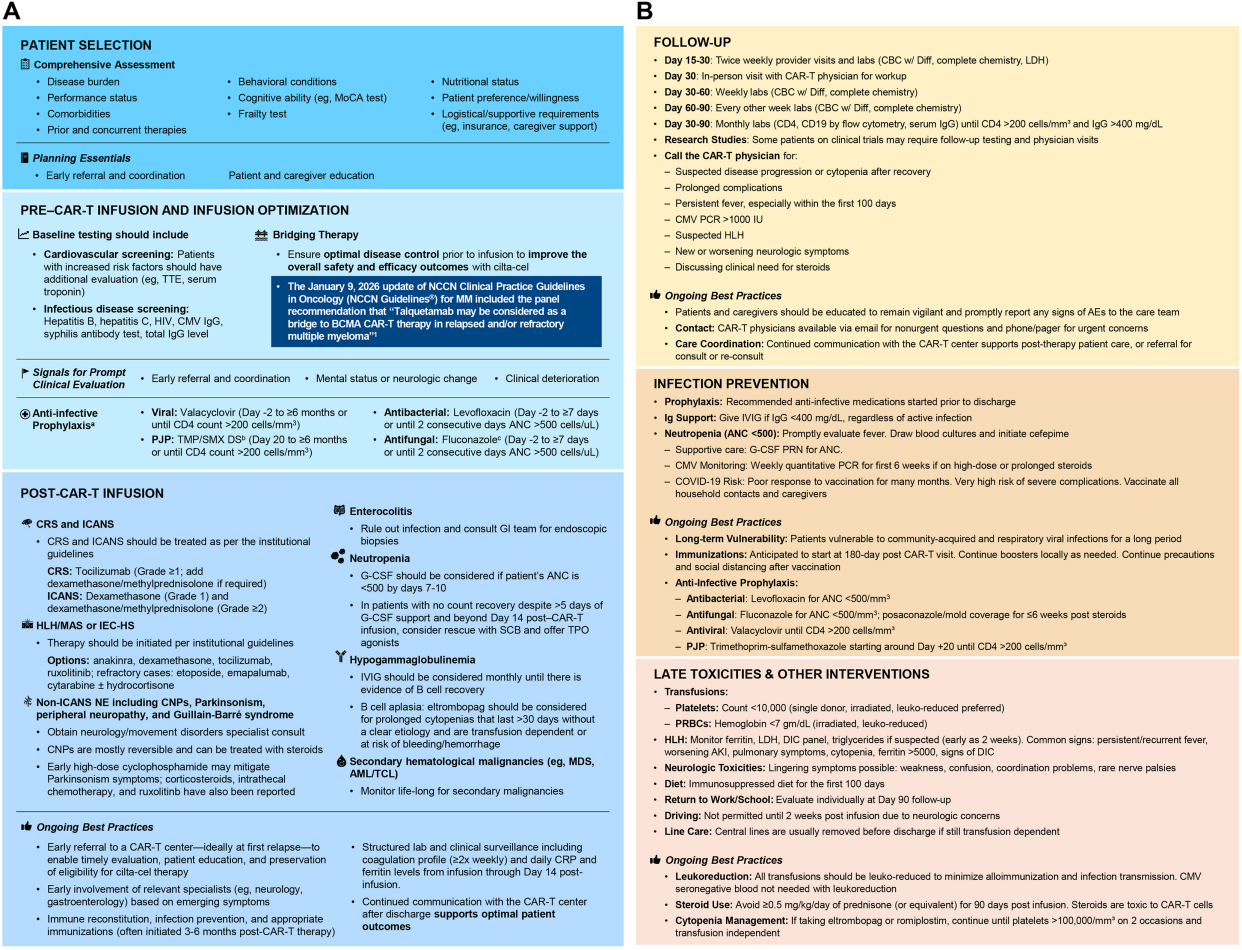
**(A)** Key considerations across the cilta-cel treatment journey. 1. Referenced with permission from the NCCN Clinical Practice Guidelines in Oncology (NCCN Guidelines^®^) for Multiple Myeloma V.5.2026. © National Comprehensive Cancer Network, Inc. 2026. All rights reserved. Accessed January 14, 2026. To view the most recent and complete version of the guideline, go online to NCCN.org. NCCN makes no warranties of any kind whatsoever regarding their content, use or application and disclaims any responsibility for their application or use in any way. ^a^Adjusted for renal function. ^b^Dapsone if transfusion dependent. ^c^Posaconazole if high risk; high risk: defined as leukemia, recent allogeneic transplant, prior history of mold infection, neutropenia >14 days, receiving >3 days of steroids or patients with HLH. Posaconazole could be continued for at least 1mo after completion of steroids. It should not be stopped if ANC <1 K/mL. **(B)** Post CAR-T outpatient follow-up and monitoring schedule after cilta-cel infusion. AE, adverse event; AKI, acute kidney injury; AML, acute myeloid leukemia; ANC, absolute neutrophil count; BCMA, B-cell maturation antigen; CAR-T, chimeric antigen receptor T-cell therapy; CBC, complete blood count; CD4/19, cluster of differentiation 4/19; cilta-cel, ciltacabtagene autoleucel; CMV, cytomegalovirus; CNP, cranial nerve palsy; COVID, coronavirus disease; CRP, c-reactive protein; CRS, cytokine release syndrome; DIC, disseminated intravascular coagulation; Diff, differential; DS, double strength; G-CSF, granulocyte colony-stimulating factor; GI, gastrointestinal; HIB, Haemophilus influenzae type B; HIV, human immunodeficiency virus; HLH, haemophagocytic lymphohistiocytosis; ICANS, immune effector cell-associated neurotoxicity syndrome; IEC-HS, immune effector cell-associated hemophagocytic lymphohistiocytosis-like syndrome; IgG, immunoglobulin G; IU, international unit; IVIG, intravenous immunoglobulin; LDH, lactate dehydrogenase; MAS, macrophage activation syndrome; MDS, myelodysplastic syndrome; MM, multiple myeloma; MoCA, Montreal Cognitive Assessment; NCCN, National Comprehensive Cancer Network; NE, neurologic event; PCR, polymerase chain reaction; PJP, *Pneumocystis jirovecii* pneumonia; PRBCs, packed red blood cells; PRN, as needed; SCB, stem cell boost; TCL, T-cell lymphoma; TMP/SMX, trimethoprim–sulfamethoxazole; TPO, thrombopoietin receptor agonist; TTE, transthoracic echocardiogram; w/, with.

Identifying eligible patients remains one of the key challenges for both academic CAR-T centers and referring community practices (21, 22). Our institution employs a structured yet flexible patient selection paradigm designed to maximize safety while ensuring equitable access to cilta-cel (**Figure 2A**). We conduct a comprehensive assessment of disease burden, comorbidities, cognition, neurologic evaluation, and patient frailty status to determine eligibility. This also proactively identifies potential challenges and anticipates AEs requiring management before and after therapy. Although comorbidities can affect apheresis success and manufacturing feasibility, evidence suggests that even patients with significant comorbidity burdens who would have been excluded from trials can have favorable outcomes with apheresis and cilta-cel (18, 23). The healthcare team then evaluates the patient’s eligibility for CAR-T therapy and provides the information to the patient/caregiver to allow for an informed treatment decision. The Foundation for the Accreditation of Cellular Therapy (FACT) guidance is used for pre-collection evaluation viral testing (24). Our standard panel includes testing for hepatitis B, hepatitis C, human immunodeficiency virus, and cytomegalovirus immunoglobulin.

Following apheresis, BT is administered, particularly for patients with rapid disease progression, high-risk factors, or extramedullary disease, to limit progression and reduce tumor burden (25, 26). BT is individualized based on patient needs, guided by several factors, including disease characteristics, treatment history, and risk of AEs (5, 27, 28). Effective BT can enhance the effectiveness of CAR-T therapy and reduce risk of toxicities, including cytokine release syndrome (CRS), immune effector cell (IEC)–associated neurotoxicity syndrome (ICANS), prolonged cytopenias, and non-ICANS neurologic events (NE), such as parkinsonism (5, 26, 29). In CARTITUDE-4, patients who achieved deeper responses to BT (≥partial response [PR]) experienced longer PFS and OS following cilta-cel infusion, with no reported cases of parkinsonism (29). By contrast, patients with suboptimal responses to BT were more likely to develop fatal infections, prolonged thrombocytopenia and neutropenia, and higher rates of non-relapse mortality after receiving cilta-cel (29). BCMA-targeted therapies are generally not recommended as BT immediately before CAR-T therapy due to the potential risk of antigen downregulation. Alternative therapies, such as immunomodulatory drugs and anti-CD38 antibodies, are preferred with an appropriate washout period, as they may enhance T-cell function and CAR T-cell activity (28). In our institutional experience, prior BCMA-targeted therapy exposure was uncommon, reflecting our approach of treating patients earlier in the disease course. In the rare cases where prior BCMA exposure occurred, there was a washout period of 3–6 months prior to cilta-cel infusion. Real-world BT experience supports clinical observations; patients who responded to BT demonstrated higher response rates and lower toxicity; and the use of talquetamab as BT was associated with lower rates of high-grade CRS/ICANS, with limited neurotoxicity (30–32). Collectively, these findings support optimizing disease control with BT prior to cilta-cel infusion.

Immediately prior to CAR-T infusion, lymphodepleting chemotherapy (fludarabine plus cyclophosphamide) creates a favorable cytokine milieu that supports CAR-T expansion and persistence (33). When indicated, lymphodepletion dosing may be adjusted for renal function without adversely affecting CAR-T outcomes (34). The CAR-HEMATOTOX score, calculated prior to lymphodepletion, enables early risk stratification for severe hematotoxicity following CAR-T therapy (35). We coordinate supportive measures (nutritional support, psychosocial counseling, and caregiver/family education) to optimize pre-infusion patient stability, maximizing their tolerability and recovery. The patients should also receive anti-infective prophylaxis prior to CAR-T therapy (**Figure 2A**).

Pre-infusion assessments focus on identifying comorbidities and infection risks that could complicate treatment. Cardiac evaluation, neurologic assessments, and infectious disease screening are essential components of pre-infusion risk mitigation.

## Infusion and acute toxicity management

The acute phase following cilta-cel infusion (Days 0–14) requires vigilant daily monitoring to identify and manage CRS, ICANS, and hemophagocytic lymphohistiocytosis/macrophage activation syndrome (HLH/MAS). The incidence of CRS and ICANS was 95% and 23% in CARTITUDE-1 and 78% and 7% in CARTITUDE-4, respectively (36). Notably, cilta-cel is characterized by a delayed CRS and ICANS onset (≈7–8 days and 7 days, respectively) compared with other CAR-T therapies (occur within the first 1–3 and 2–3 days, respectively) (37). This delayed toxicity profile has supported outpatient administration in appropriately selected patients (37). Laboratory surveillance includes daily C-reactive protein, absolute lymphocyte count (ALC^peak^), and ferritin levels and twice-weekly coagulation profiles. Any deviation from baseline mental status, hemodynamics, or organ function triggers immediate escalation. CRS is primarily managed with tocilizumab (Grade ≥1; adding dexamethasone/methylprednisolone, as needed); ICANS is managed with dexamethasone (Grade 1) and methylprednisolone (Grade ≥2) (38).

Our institution employs the American Society for Transplantation and Cellular Therapy (ASTCT) grading scales and management algorithms for CRS and ICANS, adapted to cilta-cel–specific characteristics. HLH is a hyperinflammatory syndrome associated with progression or new onset of cytopenias, coagulopathy with hyperfibrinogenemia, and/or transaminitis. In the CARTITUDE-1 and CARTITUDE-4 trials, HLH was reported in 1% (3/285) of patients (36). Given the high mortality of untreated HLH (36), we maintain a low threshold for evaluation and rapidly evaluate diagnostic markers (rapidly rising ferritin levels ≥10,000 ng/mL required) and initiate treatment without delay when HLH is diagnosed or suspected. Management of severe CRS/HLH includes anakinra, ruxolitinib, and emapalumab (38).

Early hematologic side effects (Days 0–30) reflect an expected nadir from fludarabine/cyclophosphamide lymphodepletion, often exacerbated by high-grade CRS and elevated cytokines. Neutrophil recovery follows 1 of 3 patterns: quick, intermittent biphasic, or prolonged aplastic recovery, with the latter associated with a high infection risk. Persistent neutropenia warrants evaluation for alternative causes (drug−induced myelosuppression, vitamin deficiencies, viral infections), assessing for HLH when ferritin is rapidly rising, and bone marrow assessment in severe/refractory cases. Granulocyte colony-stimulating factor (G-CSF) is the mainstay of management for early hematologic toxicity (35).

Although the risk of CRS and ICANS necessitate vigilant monitoring, most events follow predictable timelines and are manageable using established toxicity management algorithms. In our institutional experience, outpatient administration of cilta-cel is feasible and safe in carefully selected patients, effectively reducing inpatient resource utilization. Successful implementation required a robust programmatic infrastructure, including daily provider evaluations (Days 1-7) followed by biennial assessments until Day 14, alongside clearly defined escalation pathways for toxicity management and close multidisciplinary coordination. Patients are equipped with home monitoring kits (blood pressure cuffs, pulse oximeters, and thermometers) to facilitate early recognition of evolving toxicities and prompt clinical communication. Comprehensive education for both patients and caregivers are equally critical, utilizing structured counseling and standardized handouts to reinforce monitoring and emergency protocols. Strategic patient selection remains a cornerstone of safe outpatient delivery. At our center, eligibility requires a reliable 24-hour caregiver for at least 2 weeks and residence within 30 minutes of the facility. Patients with significant comorbidities requiring intensive inpatient monitoring are typically excluded from outpatient treatment. The primary indications for hospital admission during the first 2 weeks post infusion are development of fever or any grade of ICANS, consistent with our institutional escalation protocols. Of 51 outpatients, 2% (1/51) required ICU-level admission. While some centers utilize preemptive admission around Day +5 to manage anticipated cilta-cel toxicities, our program maintains a fully outpatient strategy with validated pathways for rapid inpatient transition when clinically indicated. This reproducible framework, adapted from established outpatient CAR-T models and refined through institutional experience, supports safe outpatient delivery while ensuring immediate access to higher levels of care when needed (37, 39–41).

## Post-infusion monitoring and long-term care

Following resolution of early toxicities, subacute and long-term monitoring focuses on late immune-mediated effects, neurologic complications, cytopenias, and secondary neoplasms. Our institutional approach emphasizes frequent evaluation, structured laboratory surveillance, multidisciplinary collaboration, and early intervention (**Figures 2A, B**), consistent with clinical experience and the cilta-cel prescribing information (36).

Immune-effector cell–associated enterocolitis, which manifests as watery non-bloody diarrhea, abdominal pain, tenesmus, fever, or fatigue, has been reported in both clinical trials and postmarketing settings (36, 42). We maintain vigilance for new or worsening gastrointestinal symptoms. Management includes stool studies, endoscopies, biopsies, gastroenterology consultation, systemic steroids, intravenous immunoglobulin (IVIG), and ruxolitinib/infliximab/vedolizumab for refractory cases (38, 43).

Cilta-cel has also been associated with non-ICANS NE, including cranial nerve palsies (CNP), peripheral neuropathy, and movement or neurocognitive disorders/parkinsonism. These complications may emerge from weeks to months post infusion. Parkinsonism (CARTITUDE-1, 6%, vs CARTITUDE-4, 1%) presenting as tremors, rigidity, bradykinesia, and gait abnormalities have also been reported with cilta-cel (36). These manifestations may initially present as subtle, non-specific signs (e.g., severe fatigue or cognitive/personality changes observed by caregivers) prior to the emergence of characteristic motor symptoms (44). Early recognition and prompt neurology referral are essential for new neurologic symptoms. Management of parkinsonism includes IVIG and methylprednisolone; in some cases, cyclophosphamide and ruxolitinib have been helpful (38). CNP (facial paralysis/numbness; CN VII most commonly affected; CARTITUDE-1, 3%, vs CARTITUDE-4, 9%) are predominately reversible and evaluated with magnetic resonance neuroimaging, CSF analyses, and infections workup (36, 45). Management typically includes corticosteroids, IVIG, supportive therapy, and multidisciplinary rehabilitation (38).

We monitored ALC^peak^, a surrogate for CAR-T expansion, daily during the acute monitoring period. Peak CAR-T expansion following cilta-cel occurs at a median of 14 days, after the onset of CRS/ICANS, but before the delayed onset of CNP, parkinsonism, and IEC-EC (43, 44, 46, 47). Many centers have adopted risk-adapted strategies to mitigate non-ICANS NE by intervening early to limit excessive CD4 CAR-T expansion before overt toxicity develops (43). The use of prophylactic dexamethasone in cilta-cel–treated patients with ALC levels ≥3x10^9^/L remain under investigation (43, 48).

Long-term follow-up focuses on immune reconstitution, infection prevention, hematologic recovery, and surveillance for secondary complications. IEC-associated hematotoxicity is the most common CAR-T–related AE, with prolonged cytopenias being a frequent and sometimes severe long-term toxicity that increases the risk of infection and transfusion dependence (35, 36). Persistent cytopenia (CARTITUDE-1 and CARTITUDE-4: ≥Grade 3 [not resolved by Day 30], 62% [176/285]) prompts evaluation with a bone marrow biopsy to assess relapse or other myeloid malignancies, viral reactivation, or immune dysregulation (36). When warranted, thrombopoietin receptor agonists or stem cell boosters are used to good effect (35). Post-infusion infections are the leading cause of non-relapse mortality, with viral infections predominating after Day 30 (49). Across CARTITUDE-1 and CARTITUDE-4, infections occurred in 57% (163/285) of patients (Grade ≥3, 24% [69/285]), and hypogammaglobulinemia was reported in 94% (267/285) of patients (36). Anti-infective prophylaxis and monthly monitoring of Ig levels is critical, with IVIG administered for IgG <400 mg/dL, or <600 mg/dL for patients with recurrent infections (36, 38).

As CAR-T therapies carry a recognized risk of secondary hematologic malignancies (36), we maintain ongoing vigilance for therapy-related myeloid neoplasms, including myelodysplastic syndrome and acute myeloid leukemia. In the CARTITUDE-1 and CARTITUDE-4 trials, myeloid neoplasms were reported in 5% (13/285) of patients, with a median time to onset of 447 days (range, 56–870 days) (36). These findings underscore the importance of long-term hematologic surveillance following cilta-cel. Monitoring consists of periodic complete blood counts, marrow evaluation when indicated, and hematology consultation for any abnormalities.

Physical therapy, occupational therapy, cognitive rehabilitation, and psychosocial support are essential for long-term recovery. Patients experiencing non-ICANS NE may need prolonged rehabilitative support and outpatient neurology follow-up. Ongoing communication with community providers ensures continuity as patients transition back to local care, supports early recognition of late complications, and facilitates efficient re-referral.

## Outcomes and lessons learned

Our institutional experience demonstrates that favorable clinical outcomes with cilta-cel can be achieved when supported by rigorous multidisciplinary coordination and adherence to well-defined care pathways. Our patient-reported outcomes align with real-world reports showing high response rates, long-term survival, and manageable safety with appropriate monitoring and early intervention.

Operational successes—seamless cross-service communication and consistent toxicity-management—have contributed to positive patient outcomes. Staff familiarity with CAR-T–specific toxicities and robust escalation pathways have been essential for mitigating severe CRS, ICANS, and non-ICANS NE. Key best practices include:

- Early referral pathways from community settings to ensure timely evaluation, reduce disease-related complications, and support logistic needs
- Comprehensive baseline assessments (including baseline evaluation by neurologist) to reduce the risk of unanticipated complications
- Structured monitoring and rapid intervention algorithms during the acute and post-acute phases to prevent escalation to high-grade toxicities
- Proactive recognition and management of non-ICANS NE, supported by patient and caregiver awareness, longitudinal neurologic monitoring, and early neurology referral, to facilitate timely intervention and reduce morbidity
- Long-term follow-up focused on immune reconstitution, infection prevention, and functional recovery to restore wellness and QoL

Collectively, coordinated strategies help ensure that the transformative potential of cilta-cel, observed in clinical trials, is translated into real-world benefit for a diverse patient population.

## Discussion

The integration of cilta-cel into real-world clinical practice continues to highlight both its transformative therapeutic potential and the operational complexities of delivering CAR-T therapy outside controlled trial environments. Since >50% of patients in a real-world setting are not eligible for registrational trials (18), our institutional experience and emerging clinical evidence reinforce that optimal cilta-cel outcomes are achieved through structured, anticipatory, multidisciplinary patient evaluation, preparation, and toxicity management.

A critical determinant of real-world success is effective tumor burden reduction through BT, which has been associated with improved PFS and a more favorable safety profile in clinical trials (44, 50). In CARTITUDE-4, achieving ≥PR to BT was associated with longer PFS and OS after cilta-cel, with no cases of parkinsonism (29).

The data from the CARTITUDE-4 study underscore the benefits of cilta-cel in earlier treatment lines. Cilta-cel significantly improved PFS (HR, 0.29; 95% CI, 0.22–0.39; *P* < 0.0001) and OS (HR, 0.55; 95% CI, 0.39–0.79; *P* = 0.0009) compared with SOC in patients with lenalidomide-refractory MM after ≥1 prior LOT (median follow-up, 33.6 months) (13). Cilta-cel was also associated with a longer time to worsening of myeloma-related symptoms and functional impairment (51). Notably, safety outcomes are more favorable when cilta-cel is used earlier in the treatment course. CARTITUDE-4 demonstrates lower rates of cytopenias, CRS, and parkinsonism compared with those observed in the later-line CARTITUDE-1 population (9). Our experience aligns with these findings as our patients tend to receive CAR-T in earlier treatment lines. These observations support a shift toward earlier consideration of CAR-T therapy in eligible patients. Long-term follow-up from CARTITUDE-1 suggests that cilta-cel may have curative potential, with a subset of patients achieving deep and durable remissions years after a single infusion (7). These results reinforce the importance of optimizing real-world care pathways to ensure that patients can safely reach infusion and benefit from this potentially life-altering therapy.

Our institutional experience emphasizes the essential role of early referral from community oncology practices to academic CAR-T centers. Timely referral enables effective patient education and informed decision-making, optimization of treatment in patients with comorbidities, coordination of apheresis, implementation of effective bridging strategies, and proactive management of potential toxicities before they compromise eligibility.

Adaptation of these workflows for hybrid shared-care models, where academic centers perform infusion and community partners provide long-term monitoring, are essential for broadening access as CAR-T therapy expands. Clear communication pathways, standardized toxicity management algorithms, and structured referral processes are essential components of successful hybrid models. These models must intentionally address the limited access faced by rural and minority populations through improved outreach and financial support strategies to ensure the potential of cilta-cel is realized equitably across all practice settings.

With the removal of the Risk Evaluation and Mitigation Strategy (REMS) program for cilta-cel, monitoring practices continue to evolve (52). While this regulatory change reduces administrative burden and may improve access, it places greater responsibility on treating centers and community providers to maintain vigilance for delayed toxicities. Accordingly, the primary treatment center should proactively provide referring clinicians with comprehensive guidance regarding anticipated toxicities, monitoring parameters, and escalation thresholds. Establishing standardized, bidirectional communication pathways ensure timely coordination and rapid re-engagement of the treating center, should complications arise.

Looking ahead, continued expansion of cilta-cel in clinical practice will depend on data sharing and prospective quality frameworks across institutions. Real-world evidence networks, multicenter registries, and consensus-driven quality metrics will be crucial for refining and improving eligibility criteria, toxicity prediction, bridging strategies, and long-term monitoring practices. As the field advances, institutional learning will remain central to ensuring that the potential of cilta-cel is realized equitably and safely across diverse practice settings. Emerging evidence suggests that structured remote patient monitoring may enhance safety during the early postinfusion period (53, 54). Wearable devices, digital symptom tracking tools, and scheduled telemedicine assessments may facilitate earlier identification of CAR-T associated AEs.

Our experience demonstrates that optimizing outcomes with cilta-cel requires proactive patient identification, early referral to a CAR-T center, effective tumor-burden control, structured multidisciplinary workflows, and persistent vigilance throughout the treatment process. By incorporating these elements, centers can improve the likelihood that the benefits demonstrated in CARTITUDE-4 and CARTITUDE-1 are reflected in routine practice.

## Data Availability

The original contributions presented in the study are included in the article/supplementary material. Further inquiries can be directed to the corresponding author.
